# Association Between Dental Caries and Concentration of Selected Minerals in Drinking Water Among Mongolian Adolescents: A Cross-Sectional Study With Nationwide Calcium, Magnesium, and Chloride Analyses and Regional Fluoride Analysis

**DOI:** 10.7759/cureus.117916

**Published:** 2026-10-14

**Authors:** Batbold Gan-Ochir, Enkh-Orchlon Batbayar, Khongorzul Tsogtsaikhan, Ganbaatar Yunden, Nyamdelger Bat-Orshikh, Ser-Od Khuyagaa, Batzorig Bayartsogt, Oyunsuren Enebish, Delgertsetseg Jargaltsogt

**Affiliations:** 1 Department of Oral and Maxillofacial Surgery, School of Dentistry, Mongolian National University of Medical Sciences, Ulaanbaatar, MNG; 2 Department of Operative Dentistry, School of Dentistry, Mongolian National University of Medical Sciences, Ulaanbaatar, MNG; 3 Department of Dental Hygiene, School of Dentistry, Mongolian National University of Medical Sciences, Ulaanbaatar, MNG; 4 Department of Epidemiology and Biostatistics, School of Public Health, Mongolian National University of Medical Sciences, Ulaanbaatar, MNG; 5 Department of Policy Planning, Ministry of Health of Mongolia, Ulaanbaatar, MNG

**Keywords:** adolescents, calcium, dental caries, dmf index, drinking water, fluoride, magnesium, mongolia

## Abstract

Introduction: Dental caries remains common among Mongolian children, but the relationship between the mineral composition of local drinking water and caries in adolescents has not been well characterized.

Methods: This nationwide cross-sectional study linked oral health screening records for Mongolian adolescents aged 13-17 years with area-level water quality data. The primary national analysis included 92,776 participants from 323 geographic areas with calcium, magnesium, and chloride data. Generalized estimating equations with exchangeable working correlation, clustered by geographic area, estimated adjusted associations with decayed, missing, and filled teeth (DMFT) and caries experience (DMFT >0), controlling for age, sex, and macroregion. A pre-specified supplementary fluoride analysis included 75,448 participants from 217 areas in 16 provinces/municipality where fluoride data were available.

Results: In the primary analysis, mean age was 14.59 (SD 1.31) years, mean DMFT was 1.87 (SD 2.47), and 59.24% had caries experience. Chloride was significantly associated with DMFT (B (unstandardized regression coefficient) per 10 mg/L = 0.014, 95% CI 0.004 to 0.024; p=0.006) and caries experience (adjusted OR=1.015, 95% CI 1.007 to 1.022; p<0.001) but with a small magnitude. Calcium and magnesium were not significantly associated with either outcome. In the restricted fluoride analysis, fluoride was weakly inversely associated with DMFT (B per 0.1 mg/L = -0.009, 95% CI -0.017 to -0.001; p=0.027) and caries experience (adjusted OR=0.991, 95% CI 0.983 to 0.999; p=0.021).

Conclusions: Area-level drinking water mineral concentrations were associated with dental caries outcomes among Mongolian adolescents. The fluoride result is limited to areas with available fluoride measurements, and the cross-sectional, ecological exposure design precludes causal inference.

## Introduction

Oral diseases affect a large proportion of the global population and create substantial health and economic burdens [1,2]. Untreated caries in permanent teeth has consistently ranked among the most prevalent health conditions worldwide [3]. Dental caries is a biofilm-mediated, sugar-driven, multifactorial, dynamic disease in which pathological and protective factors are in balance [4].

Dietary free sugars, fluoride exposure, oral hygiene, access to dental care, and social conditions are important determinants of caries [5]. Regular toothbrushing with fluoride toothpaste is an effective preventive measure, but individual fluoride exposure varies by product use and concentration [6]. In Mongolia, earlier research documented substantial caries experience and socioeconomic inequalities among children, highlighting the importance of population-level determinants alongside individual oral-health behaviors [7].

Drinking water may provide sustained exposure to fluoride and other dissolved minerals. Community water fluoridation remains an important preventive strategy, although contemporary evidence should be interpreted in light of differences in study designs and settings [8]. Public-health recommendations support an optimal fluoride concentration in community water systems for caries prevention [9,10]. Contemporary observational studies in the United States and England also report associations between community water fluoridation and caries outcomes in children and adolescents [11,12].

In a Danish population study of 52,057 15-year-old students, fluoride and calcium in drinking water were independently associated with lower decayed, missing, and filled teeth (DMFT) numbers [13]. A subsequent Danish analysis reported that fluoride, calcium, and family income were each associated with caries, emphasizing the importance of social context when interpreting water quality associations [14]. A further Danish ecological study found that water fluoride and maternal education explained a substantial proportion of inter-municipality variation in adolescent DMFT [15]. Evidence concerning other dissolved minerals is less consistent. In China, low-mineral direct drinking water used in schools was associated with higher dental caries incidence among schoolchildren [16]. In vitro work published in 2022 showed remineralization of artificially induced enamel lesions after exposure to mineral-rich Zamzam water; this result supports biological plausibility but does not establish population-level protection [17].

Mongolia has wide geographic variation in water minerals, high caries prevalence, and limited fluoride data. Previous evidence on water minerals and caries is limited and inconsistent. National oral health screening data provide an opportunity to assess whether area-level concentrations of fluoride, calcium, magnesium, and chloride in water are associated with caries experience in adolescents with permanent dentition. Therefore, this study had two distinct analytical components: (i) The primary national analysis conducted across all provinces and the capital city assessed calcium, magnesium, and chloride and tested the hypothesis that calcium and magnesium would be inversely associated, and chloride positively associated, with caries experience. (ii) The pre-specified supplementary fluoride analysis was restricted to 16 provinces with obtainable fluoride measurements and tested the hypothesis that fluoride would be weakly inversely associated with caries experience.

## Materials and methods

Study design and setting

This nationwide cross-sectional analytical study used secondary oral health examination data collected through the Mongolian Ministry of Health early detection, screening, and diagnostic program [18] between May 1, 2022, and December 1, 2024. The present study did not involve direct recruitment or new data collection from participants; individual demographic and oral health examination data were extracted from the existing national screening database. The original oral health data were generated through face-to-face clinical examinations conducted as part of the nationwide screening program. The program covered Ulaanbaatar and all 21 provinces of Mongolia, including 321 soums (a second-level administrative division in Mongolia). The present analysis linked adolescent examination records to routinely available drinking water quality records at the smallest shared geographic level (aimag/soum or district, hereafter geographic area). The report was prepared according to the Strengthening the Reporting of Observational Studies in Epidemiology (STROBE) statement [19].

Participants

According to the official 2024 Ministry of Health report, the source database contained dental examination records for 563,137 children aged nine months to 17 years who participated in the screening program [18]. After data cleaning and exclusion of records with missing, incomplete, or invalid information relevant to the present analysis, 540,199 children were included in the final analytical sample. No a priori sample size calculation was performed because this was a secondary analysis of an existing national database and the study aimed to include all eligible records available during the study period rather than recruit a predetermined sample. Accordingly, a complete-enumeration approach was used, whereby all records meeting the eligibility criteria were considered for inclusion. This analysis focused on adolescents aged 13-17 years with permanent dentition. Records were considered eligible when age, sex, geographic area, and DMFT were available and could be linked to water quality data. The primary calcium-magnesium-chloride analysis included 92,776 adolescents from 323 geographic areas. The supplementary fluoride analysis was restricted to 75,448 adolescents from 217 geographic areas in 16 provinces/municipality because fluoride measurements were unavailable for the remaining geographic areas (Figure 1).

**Figure 1:**
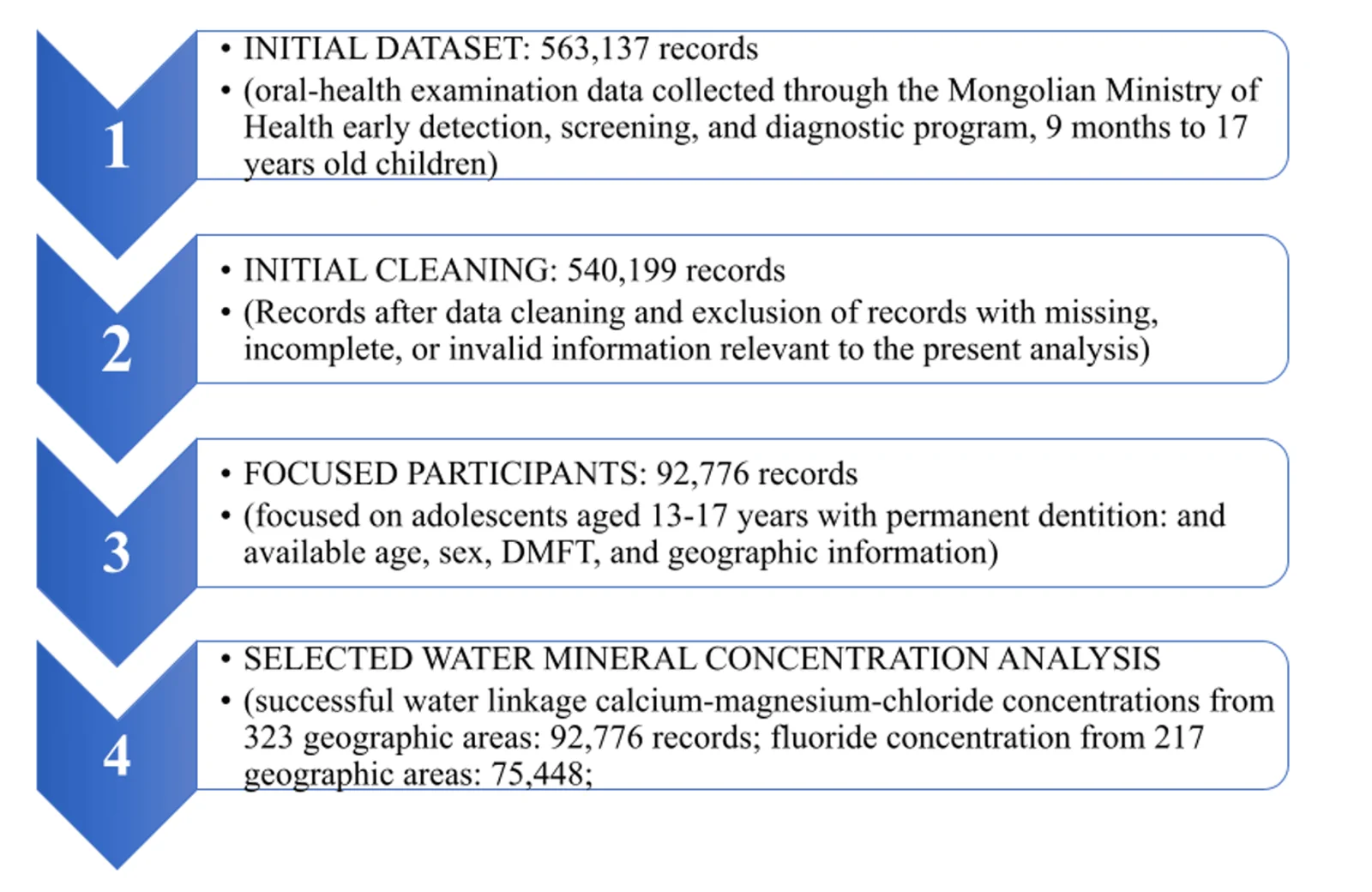
Flow diagram of study participants DMFT, decayed, missing, and filled teeth.

Dental caries assessment and outcomes

Clinical oral examinations were performed by family physicians and dentists who had received prior training within the national screening program using World Health Organization (WHO) criteria for oral health surveys [20]. Dental caries experience was assessed using the DMFT index originally described by Klein et al. [21]. For permanent dentition, the DMFT score was calculated as the sum of decayed teeth (D), teeth missing due to caries (M), and filled teeth (F): DMFT = D + M + F. A DMFT score of 0 indicated no recorded caries experience, whereas a score greater than 0 indicated the presence of caries experience; higher DMFT values represented greater cumulative caries experience. The primary outcome was individual DMFT score. The secondary outcome was caries experience, coded as caries-affected when DMFT was greater than zero and caries-free when DMFT was zero.

Drinking water mineral data and geographic linkage

Area-level drinking water quality data for 2020-2023 were obtained from routine monitoring records maintained by the National Agency for Meteorology and Environmental Monitoring and the National Center for Public Health, Mongolia [22]. Fluoride, calcium, magnesium, and chloride concentrations were expressed in mg/L, as used in international drinking water quality guidance [23], and linked to adolescent records using the shared geographic-area code. The calcium, magnesium, and chloride model was designated as the primary national analysis because these variables had coverage in all provinces/municipality. Fluoride was analyzed separately as a supplementary exposure because its records did not cover every geographic area.

Because every participant within a geographic area was assigned the same water concentration, the exposure represents area-level water composition rather than personal water intake. The data do not measure household water source, bottled water use, water consumption, or duration of residence.

Covariates

Age in years and sex were individual covariates. Macroregion (six categories) was included as a fixed-effect geographic covariate. Geographic area was accounted for through clustered analyses.

Statistical analysis

Statistical analyses were performed using IBM SPSS Statistics for Windows, version 29.0 (IBM Corp., Armonk, New York, United States). Descriptive statistics were used to summarize participant characteristics and water-mineral concentrations. Continuous variables were presented as mean±standard deviation (SD) and median (interquartile range (IQR)), as appropriate, whereas categorical variables were presented as frequency (percentage). Water mineral distributions were inspected for extreme values. Multicollinearity was assessed using variance inflation factors (VIFs) at the geographic-area level; all VIFs for fluoride, calcium, magnesium, and chloride were below 3.

Generalized estimating equation (GEE) analyses were used to account for clustering of participants within geographic areas [24]. For the continuous DMFT outcome, GEE linear regression models with a Gaussian distribution and identity link function were fitted. For caries experience (DMFT >0), GEE binary logistic regression models with a binomial distribution and logit link function were fitted to estimate adjusted odds ratios (ORs) with 95% confidence intervals (CIs). An exchangeable working correlation structure and robust standard errors were specified in all GEE models. The primary model included calcium, magnesium, chloride, age, sex, and macroregion; the supplementary model additionally included fluoride. Two-sided p-values <0.05 were considered statistically significant.

Ethics statement

Permission to use the national screening database for research purposes was granted under the Ministry of Health research framework established by Ministerial Order No. A/498 (2022). The study protocol for the secondary analysis was approved by the ethics committee of Mongolian National University of Medical Sciences, Ulaanbaatar, Mongolia (approval No. 2023/3-07/36), and only de-identified data were used for analysis.

## Results

The primary national analytical sample comprised 92,776 adolescents from 323 geographic areas. The mean age was 14.599±1.31 years, mean DMFT was 1.879±2.47, and 54,965 participants (59.24%) had caries experience. Of the total participants, 48,591 (52.37%) were female and had a higher mean DMFT and caries prevalence than male participants (Table 1).

**Table 1:** Characteristics of the primary national analytical sample and distribution accoding to sex SD, standard deviation; DMFT, decayed, missed, filled teeth.

Characteristic	Overall	Female	Male
Participants, n	92,776	48,591	44,185
Age, years, mean±SD	14.59±1.31	14.59±1.31	14.59±1.31
DMFT, mean±SD	1.87±2.47	1.98±2.50	1.76±2.43
Caries experience (DMFT>0), n (%)	54,965 (59.24)	29,414 (60.54)	25,551 (57.82)

In the primary national sample, participant-weighted mean calcium concentration was 36.88±19.32 mg/L, magnesium was 14.68±13.38 mg/L, and chloride was 36.42±79.26 mg/L. Fluoride data were available for 75,448 adolescents; within that supplementary sample, mean fluoride concentration was 0.71±0.67 mg/L (Table 2).

**Table 2:** Participant-weighted distribution of water-mineral concentrations Participant-weighted distribution means that each participant was assigned the drinking-water mineral concentration measured for their geographic area. Therefore, geographic areas with larger numbers of study participants contributed proportionally more observations to the descriptive distribution. SD, standard deviation; IQR, interquartile range.

Mineral	Frequency	Mean±SD, mg/L	Median (IQR), mg/L
Primary national analysis			
Calcium	92,776	36.88±19.32	30.78 (23.18-45.47)
Magnesium	92,776	14.68±13.38	8.08 (5.03-20.91)
Chloride	92,776	36.42±79.26	16.04 (12.06-26.33)
Supplementary fluoride analysis			
Fluoride	75,448	0.71±0.67	0.46 (0.31-0.90)

In the primary national GEE model, chloride was significantly associated with DMFT, but with a small magnitude (B (unstandardized regression coefficient) per 10 mg/L = 0.014, 95% CI 0.004 to 0.024; p=0.006). Calcium and magnesium were not significantly associated with DMFT. In the supplementary model, fluoride was weakly inversely associated with DMFT (B per 0.1 mg/L = -0.009, 95% CI -0.017 to -0.001; p=0.027), whereas chloride retained a positive association (Table 3)

**Table 3:** Adjusted generalized estimating equation analysis of associations with DMFT The dependent variable was the DMFT score, defined as the number of decayed, missing due to caries, and filled permanent teeth. Associations were estimated using  GEEs with a Gaussian distribution, identity link, exchangeable working correlation, and robust standard errors, clustering participants within geographic areas. B represents the unstandardized regression coefficient, indicating the adjusted change in mean DMFT associated with the specified increase in each predictor. CI, confidence interval; SE, standard error; DMFT, decayed, missing, and filled teeth; GEE, generalized estimating equation

Model and variable	B	Robust SE	95% CI	p value
Primary national model (n=92,776; 323 clusters)				
Calcium, per 10 mg/L	-0.003	0.012	-0.027 to 0.021	0.792
Magnesium, per 10 mg/L	-0.045	0.025	-0.093 to 0.004	0.072
Chloride, per 10 mg/L	0.014	0.005	0.004 to 0.024	0.006
Age, per year	0.170	0.022	0.127 to 0.213	<0.001
Female vs male	0.197	0.027	0.145 to 0.250	<0.001
Supplementary fluoride model (n=75,448; 217 clusters)				
Fluoride, per 0.1 mg/L	-0.009	0.004	-0.017 to -0.001	0.027
Calcium, per 10 mg/L	-0.011	0.011	-0.033 to 0.010	0.300
Magnesium, per 10 mg/L	-0.057	0.030	-0.116 to 0.003	0.061
Chloride, per 10 mg/L	0.014	0.005	0.005 to 0.023	0.002

In the primary national GEE logistic model, higher chloride concentration was statistically significantly associated with dental caries experience, although the effect size was modest (adjusted OR per 10 mg/L = 1.015, 95% CI 1.007 to 1.022; p<0.001). Calcium and magnesium were not significant. In the supplementary fluoride model, fluoride was inversely associated with caries experience (adjusted OR per 0.1 mg/L = 0.991, 95% CI 0.983 to 0.999; p=0.021), while chloride remained positively associated (Table 4).

**Table 4:** Adjusted generalized estimating equations analysis of associations with caries experience The outcome (dependent) variable was caries experience, coded as 1 for caries-affected (DMFT >0) and 0 for caries-free (DMFT = 0). Associations were estimated using generalized estimating equations (GEE) with a binomial distribution, logit link, exchangeable working correlation, and robust standard errors, clustering participants within geographic areas. Both models were adjusted for age, sex, and macroregion. The primary model included calcium, magnesium, and chloride; the supplementary model additionally included fluoride. OR, odds ratio; CI, confidence interval.

Model and variable	Adjusted OR	95% CI	p value
Primary national model (n=92,776; 323 clusters)			
Calcium, per 10 mg/L	0.984	0.959 to 1.011	0.240
Magnesium, per 10 mg/L	0.969	0.923 to 1.017	0.203
Chloride, per 10 mg/L	1.015	1.007 to 1.022	<0.001
Age, per year	1.108	1.083 to 1.133	<0.001
Female vs male	1.109	1.080 to 1.140	<0.001
Supplementary fluoride model (n=75,448; 217 clusters)			
Fluoride, per 0.1 mg/L	0.991	0.983 to 0.999	0.021
Calcium, per 10 mg/L	0.976	0.952 to 1.001	0.059
Magnesium, per 10 mg/L	0.972	0.914 to 1.033	0.362
Chloride, per 10 mg/L	1.017	1.010 to 1.023	<0.001

Sensitivity and coverage considerations

The fluoride model was restricted by the availability of fluoride data and therefore included fewer participants, geographic areas, and provinces/municipality than the primary national model (Table 5). The direction of the chloride association was similar in the primary and supplementary samples. Fluoride findings should be interpreted as associations within the 16 provinces/municipality with available fluoride records, not as estimates for all of Mongolia.

**Table 5:** Participants in the fluoride model compared to the primary model Cluster size refers to the number of participants per geographic area. The precision of area-level exposure estimates is determined primarily by the number and distribution of clusters rather than by the total individual-level sample. Ca, calcium; Mg, magnesium; Cl, chloride.

Parameter	Primary national analysis (Ca, Mg, Cl)	Supplementary fluoride analysis
Number of geographic areas (clusters)	323	217
Number of provinces/municipality	22	16
Number of participants	92,776	75,448

## Discussion

Principal findings

In this nationwide cross-sectional analysis of Mongolian adolescents, chloride concentration was positively associated with both DMFT and caries experience after adjustment for age, sex, and macroregion. Calcium and magnesium were not significantly associated with either outcome in the primary national model. In the restricted supplementary analysis, fluoride was inversely associated with both outcomes. These estimates describe associations with area-level water composition and should not be interpreted as evidence that any mineral causes or prevents caries.

Comparison with previous studies

The inverse fluoride association is directionally consistent with prior studies linking water fluoride to lower caries experience, including Danish municipality-level analyses [13-15]. It is also compatible with the broader literature on community water fluoridation, although the certainty and contemporary applicability of the evidence vary across settings [8]. Contemporary studies from the United States [11], England [12], and Brazil [25] likewise support the need to consider local context, exposure measurement, and social inequality when interpreting fluoride-related associations. The fluoride result in this study is modest and limited to geographic areas with available measurements; it should not be generalized to provinces/municipality without fluoride data.

We did not observe independent calcium or magnesium associations in the primary national model. This differs from Danish studies in which calcium and fluoride were associated with lower DMFT [13,14]. Differences in water sources, mineral distributions, background fluoride exposure, caries measurement, social conditions, and the ecological exposure assignment may account for discordant estimates. The Chinese study of low-mineral direct drinking water also suggests that mineral-depleted water may be associated with greater caries burden, but that setting involved school water systems and cannot be directly equated with the geographically assigned exposure in Mongolia [16].

The positive chloride association has no established direct cariogenic interpretation in this analysis. Chloride may be a marker of local hydrogeochemical conditions, water source, salinity, unmeasured behavioral factors, or geographic confounding. Consequently, it should be considered a hypothesis-generating finding requiring replication with more detailed water source and individual-level data. The in vitro study of mineral-rich Zamzam water offers biological plausibility for mineral effects on enamel, but it cannot validate or explain population-level associations observed here [17].

Strengths and limitations

Strengths include the large national screening dataset, standardized permanent-dentition outcome, linkage to routinely collected water-quality data, and modelling that accounted for clustering of participants within geographic areas. The primary model retained broad national coverage for calcium, magnesium, and chloride. The study also transparently separates the fluoride analysis because fluoride data were available in only 217 geographic areas in 16 provinces/municipality.

Several limitations are important. First, the cross-sectional design does not establish temporality or causality. Second, water exposure was assigned at the geographic-area level and may not represent each adolescent's household source, bottled-water use, intake, duration of residence, or cumulative exposure during tooth development. Third, unmeasured sugar intake, socioeconomic circumstances, fluoride toothpaste use, toothbrushing quality and frequency, dental-care access, and environmental factors may have produced residual confounding. Sugar exposure and fluoride toothpaste use are both important caries determinants [5,6]. Fourth, screening participation was not a probability sample of all Mongolian adolescents. Fifth, the fluoride analysis had incomplete geographic coverage and may be subject to selection related to data availability. These limitations restrict causal and individual-level interpretation. Finally, the oral examination and drinking-water data were linked based on the geographic location of residence, using the national civil registration number as the basis for linkage. Mineral concentrations of drinking water were assigned using the mean of all available measurements from 2020-2023 for the corresponding geographic area.

Implications

The findings support strengthening integrated oral-health and water-quality surveillance in Mongolia. Future research should prospectively collect repeated water measurements, identify household water sources, measure total fluoride exposure and fluoride toothpaste use, capture diet and socioeconomic conditions, and use multilevel designs that distinguish individual from area-level effects. Chloride should be evaluated with broader hydrochemical profiles rather than treated as an isolated causal exposure.

## Conclusions

Among Mongolian adolescents, higher area-level chloride concentration was associated with higher DMFT and odds of caries experience, whereas calcium and magnesium were not independently associated with the outcomes in the primary national model. In the supplementary analysis limited to areas with fluoride records, higher fluoride concentration was associated with slightly lower DMFT and odds of caries experience. These cross-sectional area-level associations warrant confirmation in studies with complete exposure coverage and individual-level behavioral and water-use information.
